# Therapeutic Hypothermia Inhibits the Classical Complement Pathway in a Rat Model of Neonatal Hypoxic-Ischemic Encephalopathy

**DOI:** 10.3389/fnins.2021.616734

**Published:** 2021-02-12

**Authors:** Tushar A. Shah, Haree K. Pallera, Cortney L. Kaszowski, William Thomas Bass, Frank A. Lattanzio

**Affiliations:** ^1^Department of Pediatrics, Eastern Virginia Medical School, Norfolk, VA, United States; ^2^Department of Microbiology and Molecular Cell Biology, Eastern Virginia Medical School, Norfolk, VA, United States; ^3^Children’s Specialty Group, Norfolk, VA, United States; ^4^Children’s Hospital of The King’s Daughters, Norfolk, VA, United States; ^5^Department of Physiological Sciences, Eastern Virginia Medical School, Norfolk, VA, United States

**Keywords:** c1q, complement – immunological term, therapeutic hypothermia, hypoxic ischemic encephalopathy, microglia, neuroinflammation

## Abstract

**Objective:**

Complement activation is instrumental in the pathogenesis of Hypoxic-ischemic encephalopathy (HIE), a significant cause of neonatal mortality and disability worldwide. Therapeutic hypothermia (HT), the only available treatment for HIE, only modestly improves outcomes. Complement modulation as a therapeutic adjunct to HT has been considered, but is challenging due to the wide-ranging role of the complement system in neuroinflammation, homeostasis and neurogenesis in the developing brain. We sought to identify potential therapeutic targets by measuring the impact of treatment with HT on complement effector expression in neurons and glia in neonatal HIE, with particular emphasis on the interactions between microglia and C1q.

**Methods:**

The Vannucci model was used to induce HIE in term-equivalent rat pups. At P10-12, pups were randomly assigned to three different treatment groups: Sham (control), normothermia (NT), and hypothermia (HT) treatment. Local and systemic complement expression and neuronal apoptosis were measured by ELISA, TUNEL and immunofluorescence labeling, and differences compared between groups.

**Results:**

Treatment with HT is associated with decreased systemic and microglial expression of C1q, decreased systemic C5a levels, and decreased microglial and neuronal deposition of C3 and C9. The effect of HT on cytokines was variable with decreased expression of pro and anti-inflammatory effectors. HT treatment was associated with decreased C1q binding on cells undergoing apoptosis.

**Conclusion:**

Our data demonstrate the extreme complexity of the immune response in neonatal HIE. We propose modulation of downstream effectors C3a and C5a as a therapeutic adjunct to HT to enhance neuroprotection in the developing brain.

## Introduction

Neonatal hypoxic ischemic encephalopathy (HIE) is a significant worldwide public health problem affecting 1–2/1000 live-births in the developed world, with as much as an eight-fold higher incidence in low and middle income countries with limited access to healthcare (Kurinczuk et al., 2010). Without treatment, mortality rates in infants with HIE range from 10 to 60% depending on disease severity, with at least 25% of survivors manifesting significant neurodevelopmental disabilities (Jacobs et al., 2013). Therapeutic hypothermia (HT) is the standard of care treatment for HIE, and while it improves survival and neurodevelopment in newborns with HIE (Shankaran, 2012), childhood outcomes after HT treatment demonstrate variable neuroprotection, with benefits waning over time (Shankaran et al., 2012). The limited success of HT may be attributable to its variable modulation of inflammatory mediators of reperfusion injury (Rocha-Ferreira et al., 2017). Understanding the effect of HT on individual immune pathways may be instrumental in developing targeted neuroprotective strategies to further improve outcomes after HIE.

**FIGURE 1:**
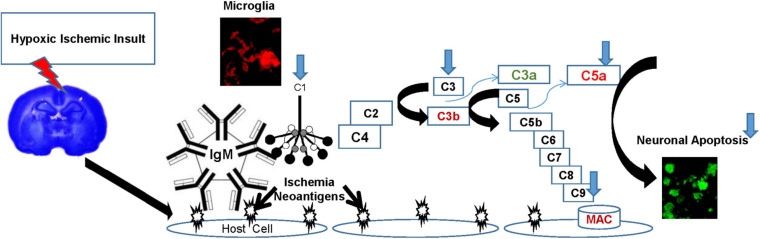
The Complement Cascade in HIE. Hypoxic insult induces expression of ischemia-induced “neoantigens” on the surface of vascular endothelial cells. These neoantigens are recognized by natural antibodies (IgM) initiating systemic classical complement pathway activation by binding C1q, characterized by a series of cleavages, culminating in the formation of a membrane attack complex (MAC) that has lytic activity. C5a and C3b are intermediates in the cascade, which execute chemotactic and phagocytic functions, respectively, while C3a has been shown to be anti-inflammatory in the acute phase. Components of the classical complement pathway (C1q, C3, and C9) are also endogenously produced in the brain, primarily in microglia after HI injury. C1q facilitates a non-inflammatory uptake of apoptotic neurons by microglia, thus limiting the exposure of surrounding neurons to toxic intracellular contents such as glutamate. Thus, microglial synthesis of C1q is a neuroprotective mechanism, and emphasizes the role of microglia and C1q in cellular homeostasis. However, in HI injuries, the extent of the damage amplifies the cascade, and mediators such as C5a perpetuate the inflammatory damage. HT (blue arrows) modulates the complement pathway in HIE by decreasing the microglial expression of C1q, C3, C9, systemic production of C5a, resulting in decreased neuronal apoptosis.

The complement system is the most potent inflammatory cascade in humans and plays a major role in innate immune defense as well as many inflammatory diseases, including ischemia-reperfusion injuries such as HIE (Arumugam et al., 2006; Liu and McCullough, 2013). C1q is the first subcomponent of the classical complement pathway and plays critical roles in health and disease. The classical complement cascade is initiated by C1q, binding to antigen-antibody immune complexes on the cell surface, or binding to specific receptors expressed during apoptosis. Once bound, C1q initiates the complement cascade characterized by a series of cleavages, leading to stepwise activation culminating in the of a membrane attack complex that has lytic activity (Figure 1). C5a and C3b are intermediates in the cascade, which execute chemotactic and phagocytic functions, respectively (Perry and O’Connor, 2008). Known C1q functions include its involvement in neuronal synapse pruning, dendritic cell maturation, immune modulation, cell differentiation, cancer progression, and pregnancy regulation (Kouser et al., 2015). While beneficial during development, unregulated complement-mediated synapse pruning can be detrimental during aging and disease (Fonseca et al., 2017). C1q binding to apoptotic cells has been shown to induce complement activation (Nauta et al., 2002). Experimental studies have shown that C1q is highly expressed in the brain following ischemia (Schafer et al., 2000), and that classical complement pathway activation via C1 generates pro-inflammatory mediators such as C5a, which are associated with HI brain injury (Rocha-Ferreira and Hristova, 2015). While deletion of C1q reduces brain infarction and neurofunctional deficit in a mouse model of neonatal HIE (Ten et al., 2005, 2010), failure to prune excessive excitatory synapses during development leads to epileptogenesis in C1q deficient mice (Chu et al., 2010). C1q enhances the uptake of apoptotic neurons and neuronal blebs by microglia, thus limiting toxic intracellular contents such as glutamate from being released, thereby preventing excitotoxic damage to surrounding neurons (Fraser et al., 2010). Traditionally considered anaphylatoxins, C3a and C5a seem to have dual roles in neuroinflammation as well (Mukherjee et al., 2008; Jarlestedt et al., 2013). C3a induces both pro- and anti-inflammatory effects that contribute to the disease phenotype. Anti-inflammatory effects of C3a dominate the acute phase of inflammation, whereas the proinflammatory aspects of the molecule dominate the chronic phase of inflammation (Coulthard and Woodruff, 2015). Similarly, in a mouse model of contusive spinal injury, C5aR antagonism in the acute phase improves recovery. However, prolonged C5aR blockade interferes with formation of reparative scar tissue, leading to a decreased ability to confine pathology, ultimately resulting in larger lesion volumes, increased spinal inflammation and poorer neurological recovery (Brennan et al., 2015).

HIE induces both a central and a systemic inflammatory response which contributes to secondary neuronal damage. The immune response is primarily initiated by activation of microglia, the major resident immune cells in the brain. Activated microglia can disrupt the blood brain barrier through the release of several inflammatory neuromodulators, resulting in infiltration of peripheral immune cells into the normally immune-privileged brain milieu, ultimately resulting in exacerbation of brain injury (da Fonseca et al., 2014; Mirza et al., 2015). C1q is predominantly produced by microglia in response to ischemia and accumulates on neuronal cells (Mack et al., 2006; Alawieh et al., 2015). Microglial synthesis of C1q is consistent with a reparative and protective role for microglia and the importance of an anti-inflammatory environment to maintain normal brain function. However, in infections and brain injuries such as HIE, the extent of the damage amplifies the complement cascade, and can potentially exacerbate the inflammatory polarization of microglia with detrimental results (Cowell et al., 2003; Fonseca et al., 2017). In a rat model of neonatal HIE, microglia were found to be the major local source of the complement component C3, an important contributor to ischemia-reperfusion injury (Cowell et al., 2003; Ma et al., 2019).

The challenge in developing new therapies for HIE is exemplified by the wide-ranging role of the complement system in neuroinflammation, homeostasis and neurogenesis. Identification of potential therapeutic targets in the complement pathway needs to be considered carefully, since an ill-timed modulation of a component could interfere with its developmental and protective role in the CNS. Understanding the effect of neuroprotective strategies such as HT on key inflammatory pathways in HIE is critical in identifying targets for therapy. The effect of HT on the complement pathway in neonatal HIE is relatively unknown. Previous work in our lab has shown that HT modulates complement activation in a complex manner in a rat model of neonatal HIE (Shah et al., 2017). Ischemic brains demonstrate significantly greater expression of C1q, C3 and C3-split products, and C9, associated with greater extent of cerebral damage (Ten et al., 2005; Shah et al., 2017). Here, we seek to measure the impact of HT on expression of key complement components in specific cell types. We speculate that therapeutic hypothermia partly mediates neuroprotection in HIE by modulating interactions between components of the classical complement pathway and microglia.

## Materials and Methods

### Materials

Primary antibodies used for assays: Goat anti-rat C3 IgG (MP Biomedicals, Santa Ana, CA, United States), Chicken anti-C3/C3a (Abcam, Cambridge, MA, United States), Rabbit anti-rat C9 (generously provided by Professor Paul Morgan, Cardiff, United Kingdom), goat anti-human C1q (Complement Technology, Inc., Tyler, TX, United States), mouse anti-NeuN (EMD Millipore, Temecula, CA, United States), chicken anti-NeuN (EMD Millipore), mouse anti-GFAP (Sigma-Aldrich, St. Louis, MO, United States), chicken anti-GFAP (Abcam), goat anti-Iba1 (Abcam), rabbit anti-C3aR (Bioss Inc., Woburn, MA, United States), Mouse anti-C5aR (Hycult Biotech., Plymouth Meeting, PA, United States). Secondary antibodies included goat anti-mouse horseradish peroxidase (HRP) (Sigma-Aldrich), goat anti-chicken IgY HRP (Genway Biotech, San Diego, CA, United States), goat anti-rabbit HRP (Sigma-Aldrich), rabbit anti-goat HRP (Thermo Fisher Scientific, Grand Island, NY, United States), biotinylated goat anti-mouse IgG (H + L), biotinylated goat anti-chicken IgG (H + L) (Vector Laboratories, Burlingame, CA, United States), biotinylated goat anti-rabbit IgG (H + L) (Bioss Inc), donkey anti-goat IgG (H + L) Alexa Fluor (AF) 488/568, goat anti-chicken IgG (H + L) AF 488, goat anti-mouse IgG (H + L) AF 568, and goat anti-rabbit IgG (H + L) AF 405/488 (Life Technologies, Grand Island, NY, United States).

### Animal Model of Unilateral Hypoxia-Ischemia

**FIGURE 2:**
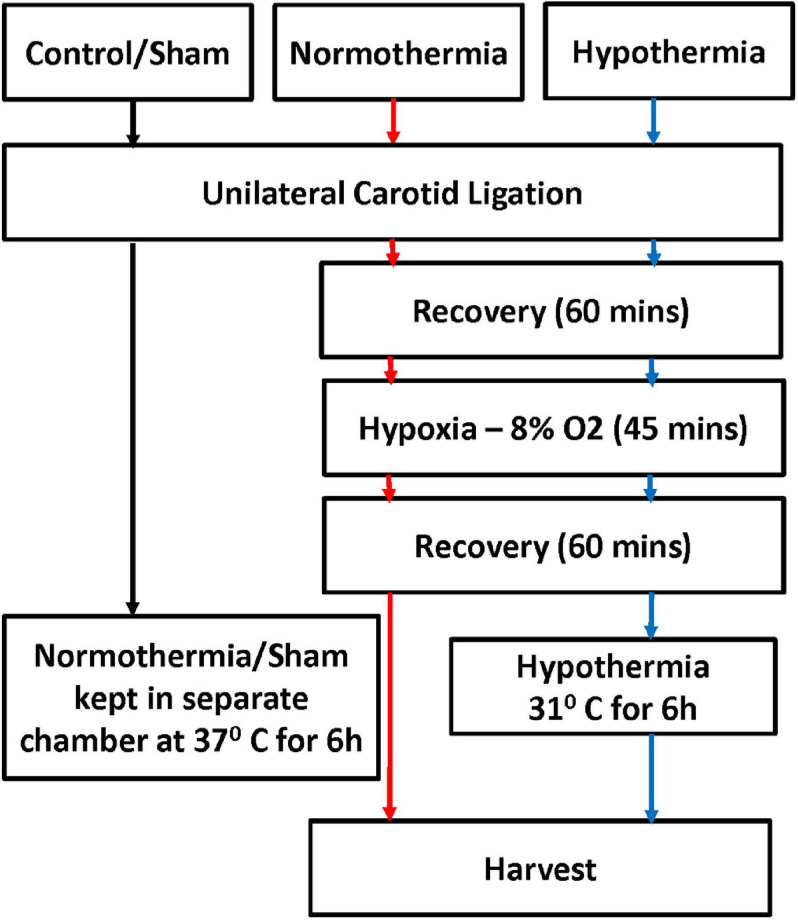
Experimental protocol. Term equivalent rat pups at P10-12 were randomly assigned to three different treatment groups: no intervention/sham (control), normothermia (NT) and hypothermia (HT). Experimental animals underwent unilateral carotid ligation followed by exposure to hypoxia (8% O_2_/balanced nitrogen, Vannucci model) for 45 min. HT animals were placed in open jars in a temperature-controlled chamber to maintain a target rectal temperature of 31–32°C for 6 h, Control, NT, and HT animals were kept in a separate chamber at 37°C. After intervention, pups were placed back with the dam, and harvested at different time points.

All animal experiments were performed under approved protocols by the Eastern Virginia Medical School (EVMS) Institutional Animal Care and Use Committee. Timed pregnant Wistar rats (Hilltop Lab Animals Inc., Scottsdale, PA, United States) were purchased at embryonic day 19, housed individually, and allowed to deliver spontaneously. Pups were randomized on the day of birth to control for litter effect (10/litter). At P10-12 (term-equivalent), pups were randomly assigned to 3 different treatment groups: Sham (control), normothermia (NT) and hypothermia (HT) treatment. As previously described (Shah et al., 2017), experimental animals underwent unilateral carotid ligation followed by exposure to hypoxia (8% O_2_/balance nitrogen, Vannucci model) for 45 min. Pups in the HT group were placed in open jars in a temperature-controlled chamber set to 28–30°C to maintain a target rectal temperature of 31–32°C for 6h, while pups in the control and NT groups were kept in a separate chamber at 37°C. After intervention, pups were placed back with the dam and harvested at different time points (Figure 2).

### Tissue Harvest and Processing

Animals were deeply anesthetized with a fatal dose of pentobarbital (Fatal-Plus^TM^). After drawing blood, animals were perfused with ice-cold PBS (followed by 10% neutral buffered formalin for perfusion fixation if being harvested for histopathology). Harvested brains were separated into right and left hemispheres and stored in liquid nitrogen until use. Complete mini EDTA-free protease inhibitor cocktail (Roche) was dissolved in homogenization buffer (300 mM sucrose, 0.05 mM CaCl_2_, 0.1 mM MgCl_2_, 0.1 mM NaHCO_3_, 1 mM Na_3_VO_4_) immediately before use and each brain lobe was homogenized on ice using a mechanical homogenizer. Whole cell protein fractions were extracted by centrifugation of the homogenate at 6000 RPM. The supernatant was collected, aliquoted and stored at −80°C until use. Protein concentration was determined using a bicinchoninic acid assay (BCA) according to the manufacturer’s recommendations (Thermo Fisher Scientific). For histopathology, 10% NBF was used for perfusion-fixation, brains were processed and paraffin embedded (Excalibur Pathology, Inc., Norman, OK, United States) and 5 μm coronal sections were cut using a RM2125 rotary microtome (Leica Microsystems). To isolate plasma, blood collected from cardiac puncture in EDTA tubes was incubated at room temperature (RT) for 45 min, incubated on ice for 45 min, centrifuged and supernatant collected, aliquoted, and stored at −80°C.

### ELISA

CNS C1q levels and systemic C1q and C5a levels were measured using a fluorescent immunoassay. For CNS C1q levels, 150 μg of whole cell brain lysate in 1 × PBS was added to the wells of a Costar high binding, black flat bottom 96-well polystyrene microplate (Corning, Steuben County, NY, United States) and incubated overnight at 4°C. The plates were washed three times with 1x PBS-0.1% Tween 20 (PBST) and blocked with 10% normal donkey serum (NDS) for 1 h. A 1:50 dilution of goat anti-human C1q (Complement Technology, Inc., Tyler, TX, United States) in NDS was added into the wells and incubated for 1 h at RT. After washing, a 1:500 dilution of donkey anti-goat Alexa Fluor (AF) 488 (Life Technologies, Grand Island, NY, United States) in NDS was added, and incubated for 1 h at RT. Wells were then washed and blocked with 10% normal goat serum (NGS) for 1 h at RT. A 1:250 dilution of mouse anti-GAPDH (Abcam, Cambridge, United Kingdom) in NGS was added into the wells and incubated for 1 h at RT. Wells were washed and a 1:500 dilution of goat anti-mouse AF 568 (Life Technologies, Grand Island, NY, United States) in NGS was added to incubate for 1 h at RT. The wells were washed and the plate read using a Synergy HT (BioTek, Winooski, VT, United States). For systemic C1q and C5a levels, 100 μl of 1:5 plasma in 1X PBS was added to the wells of a plate described above and incubated overnight at 4°C. The plates were washed three times with PBST and blocked with NDS or NGS for 1 hr. Samples were probed with either 1:50 goat anti-human C1q (Complement Technology) in NDS or 1:200 mouse anti-human C5a (Human Complement Component C5a DuoSet ELISA, R&D Systems, Inc., Minneapolis, MN, United States) in NGS for 1 h at RT. The wells were washed and 1:500 donkey anti-goat 488 (Life Technologies) in NDS or 1:500 goat anti-mouse AF 568 (Life Technologies) in NGS was added, and incubated for 1 h at RT. The wells were washed and the plate read using a Synergy HT (BioTek). Systemic C3a Levels in plasma (1:20 dilution) were measured using a standard ELISA, according to the manufacturer’s instructions (Rat C3a ELISA Kit, TSZ ELISA, Waltham, MA, United States).

### Western Blots

50 μg of whole cell lysate in 1x PBS were separated on a 4–20% gradient Mini-PROTEAN TGX precast gradient gel (Bio-Rad) under reducing conditions and transferred to a 0.2 μm Immun-Blot PVDF membrane (Bio-Rad, Hercules, CA, United States). After washing, the membrane was blocked for 1 h with 10% normal donkey serum (NDS) in PBS, followed by probing with goat anti-rat GAPDH (R&D Systems, Minneapolis, MN, United States), followed by a 1:5000 donkey anti-goat IRDye 680 secondary antibody. The membrane was blocked with 10% normal goat serum (NGS) in PBS, followed by probing with rabbit anti-C3aR (Bioss Inc., Woburn, MA, United States) or Mouse anti-C5aR (Hycult Biotech., Plymouth Meeting, PA, United States), followed by a 1:5000 goat anti-rabbit IRDye 800 secondary antibody. The membrane was imaged using a LICOR Clx Odyssey imaging system using both 680 and 800 nm fluorescent channels.

### Multiplex Plates

Rat plasma and brain homogenates were collected as previously described. All samples were kept on ice during the setup process. Protein concentration of brain homogenates was calculated using a BCA assay. Bio-Plex Pro Assay rat cytokine plates were purchased from Bio-Rad (Hercules, CA, United States). A 1:4 dilution of plasma and brain homogenate was tested following the Bio-Plex Pro cytokine plate protocol. The plates were read on the Bio-Rad MagPlex.

### Histopathology

#### 2,3,5-Triphenyl-2H-Tetrazolium Chloride (TTC) Staining

Harvested brains were sliced into 2 mm coronal sections, and incubated in the dark with 2% TTC at 37°C for 30 min. Sections were formalin fixed before digital scanning. Image J was used for analysis.

#### Cresyl Violet Staining

All washes were performed with running tap water at room temperature. Sections were deparaffinized, rehydrated, rinsed in H_2_O, and incubated in 0.1% cresyl violet solution (Sigma-Aldrich) (0.1 g cresyl violet acetate, 100 ml H_2_O, 0.3 ml glacial acetic acid) for 10 min. Sections were then washed in water, dehydrated (100%, 95%, 70% ethanol, xylene), mounted (Cytoseal XYL) and cover-slipped.

#### Multiple Label Immunofluorescence and TUNEL Staining

Five μm paraffin-embedded coronal brain sections on slides were incubated (1 h; 60°C), deparaffinized with xylene, and rehydrated with ethanol (100%, 95%, 70%; RT). Endogenous peroxidase activity was blocked (0.3% H_2_O_2_/MeOH; 10 min; RT), and heat induced antigen retrieval was performed (when indicated) using 10 mM sodium citrate, pH 6.0. 10% normal serum (from the same species as the host of the secondary antibody) was used to block and to dilute antibodies. Samples were washed twice for 10 min each, in PBST between each step.

After blocking for 1 h at RT, tissues were incubated with 1:300 goat anti-Iba1 (Abcam), 1:250 goat anti-GFAP (Abcam), 1:500 chicken anti-NeuN (Millipore, Billerica, MA, United States), or 1:100 mouse anti-NeuN (Millipore) in host appropriate blocking serum overnight at 4°C. Primary antibodies were probed with 1:500 donkey anti-goat (Abcam), goat anti-chicken (Abcam), or goat anti-mouse (Abcam) AF 488 or 568 in appropriate blocking serum for 1 h at RT. Tissues were blocked a second time with NGS for 1 h at RT. Samples were then probed with 1:300 mouse anti-C1q (Abcam), 1:300 rabbit anti-C9, 1:200 rabbit anti-TMEM (Novus Bio, Centennial, CO, United States), 1:100 rabbit anti-C3aR (Bioss), 1: 100 Mouse anti-C5aR (Hycult) or 1:200 chicken anti-C3/C3a (Abcam) in NGS overnight at 4°C and then with 1:500 goat anti-mouse, rabbit, or chicken (Abcam) AF 488 or 568 in NGS for 1 h at RT.

For C1q/TUNEL (terminal deoxynucleotidyl transferase dUTP nick end labeling) co-staining, tissue was incubated with the TUNEL solution according to manufacturer’s instructions (*In Situ* Cell Death Detection Kit, Roche Molecular Diagnostics, Pleasanton, CA, United States), followed by blocking with NGS for 1 h at RT. C1q was probed using 1:300 mouse anti-C1q (Abcam) in NGS for 30 min at 37°C. This was followed with incubation in 1:500 goat anti-mouse (Abcam) AF 568 for 30 min at 37°C. All samples were mounted with aqueous mounting media with 4′,6-diamidino-2-phenylindole (Vectashield antifade mounting medium with DAPI, Vector Laboratories, Burlingame, CA, United States) and coverslipped.

Fluorescent images of the cortex were taken using a digital camera (DP70, Olympus Center, Valley Forge, PA, United States), mounted on a BX50, Olympus microscope at 20x magnification. Adobe Photoshop CS5 was used to merge immunofluorescence images and Image J (National Institutes of Health) was used for analysis. When indicated, fluorescent images were converted to binary using Image J, and the “analyze particles” function was used to count cells.

#### Statistical Analysis

Means and standard deviation (SDs) were calculated from independent experiments. Statistical comparisons were made using the paired *t*-test and ANOVA where appropriate. All results were standardized to Sham/Control animals. Statistical analysis was performed with OpenEpi (Emory University) and SAS V9.3 (Cary, NC, United States).

## Results

### HT Is Neuroprotective in Neonatal HIE

**FIGURE 3:**
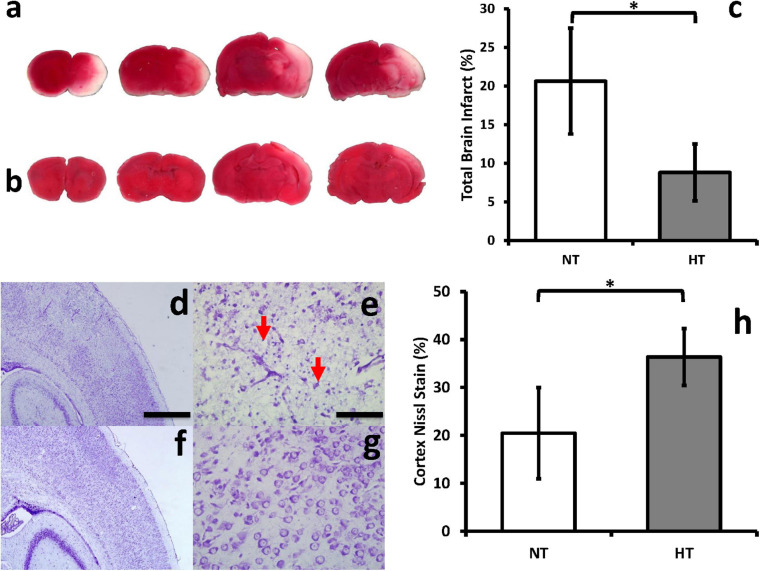
HT decreases brain infarct size and improves neuronal integrity after HIE. Brain infarct sizes were compared between groups using TTC, which stains viable portions of the brain red. Representative brain slices for NT **(a)** and HT **(b)** animals are shown. Quantification with Image J showed that HT treatment (gray bar) significantly decreased infarct size compared to NT (white bar) animals **(c)**. NT **(d,e)** animals demonstrated significantly greater neuronal disintegration and pyknosis (red arrows) when compared to HT **(f,g)** animals on cresyl violet staining. Significantly greater amount of Nissl staining in the cortex was noted in HT animals **(h)**. [*n* = 3 animals per group, cresyl violet sections depict ipsilateral hemispheres, cortex and hippocampus, 48 h after injury; Magnification – 4X **(d,f)**; 20X **(e,g)**; Scale bars: 4X images 500 μm, 20X images 100 μm; error bars denote ±SD, * denotes *P* < 0.05].

In the Vannucci rat model, we confirmed that HT treatment after HIE is neuroprotective, as demonstrated by decreased brain infarct size on TTC staining (Figures 3a,b). HT animals displayed a decrease in infarct size by an average of 57% (±4.7% SD) compared to NT animals (*P* = 0.01) (Figure 3c). Similarly, NT (Figures 3d,e) animals demonstrated significantly greater neuronal disintegration when compared to HT (Figures 3f,g) animals on cresyl violet staining. Significantly greater amount of Nissl staining in the cortex was noted in HT animals (Figure 3h).

### HT Is Associated With Decreased Local and Systemic Complement Expression in Neonatal HIE, but Effect on Cytokines Is More Variable

We measured local and systemic effects of HT treatment on the complement cascade and downstream cytokines.

#### Complement

**FIGURE 4:**
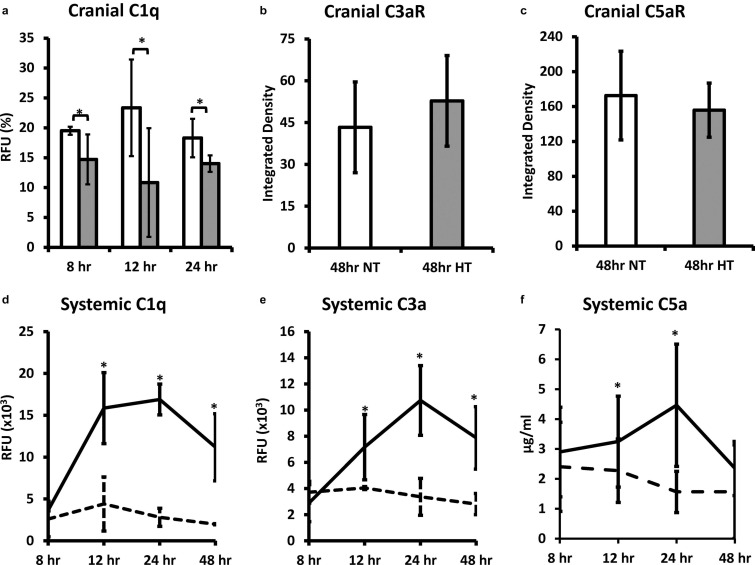
HT decreases systemic and brain complement expression in neonatal HIE. Whole brain C1q levels in NT animals were significantly higher than HT treated animals at 8, 12, and 24 h after the initial insult **(a)**. There were no significant differences in the expression of whole brain C3aR **(b)** or C5aR **(c)** between NT and HT treated groups. C1q levels in the plasma of NT animals were significantly higher than HT treated animals at 8, 12, and 24 h after injury **(d)**. Systemic C3a **(b)** and C5a **(c)** levels in NT animals were significantly higher than HT treated animals at 12 and 24 after injury. The differences in C5a persisted out to 48 h. (*n* = 7 animals per group, solid lines/white bars – NT; dotted lines/gray bars – HT; error bars denote ±SD, * denotes *P* < 0.05).

Whole brain C1q levels in NT animals were significantly higher than HT treated animals at 8, 12, and 24 h (*P* < 0.05) after the initial insult (Figure 4a). There were no significant differences in the expression of whole brain C3aR expression (receptor for C3a) or C5aR (receptor for C5a) between NT and HT treated groups (Figures 4b,c). C1q levels in the plasma of NT animals were significantly higher than HT treated animals at 8, 12, and 24 h (*P* < 0.05) after the initial insult (Figure 4d). Systemic C3a (Figure 4e) and C5a (Figure 4f) levels in NT animals were significantly higher than HT treated animals at 12 and 24 (*P* < 0.05) after the initial insult. The differences in C5a persisted out to 48 h.

#### Cytokines

**FIGURE 5:**
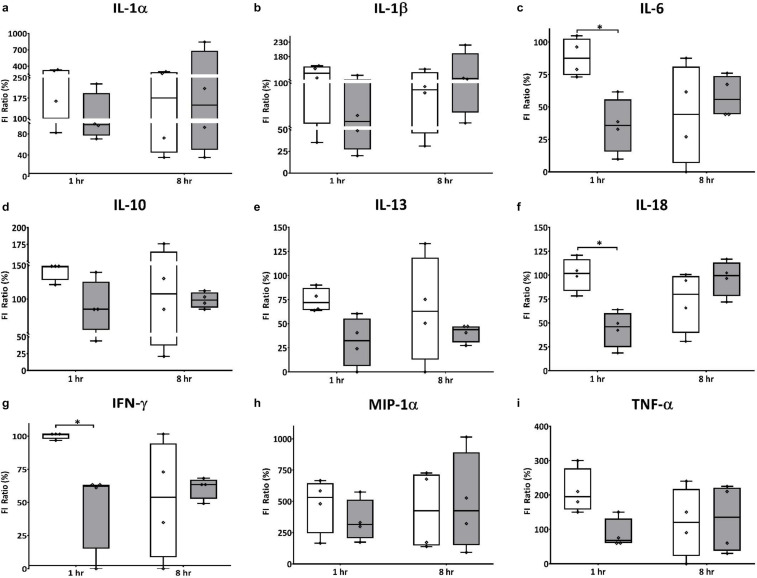
HT decreases expression of cranial pro and anti-inflammatory effectors in neonatal HIE. HT significantly decreased expression of pro-inflammatorycytokine IL-18 **(f)** 1 h after therapy was initiated, but had no effect on IL-1β expression **(b)**. While the differences did not reach statistical significance, HT also decreased expression of other pro-inflammatorycytokines IL-1α **(a)**, MIP-1α **(h)** and TNF-α **(i)** when compared to NT animals. Interestingly, HT also decreased the expression of anti-inflammatory mediators IL-10 **(d)** and IL-13 **(e)**, as well as the expression of IL-6 **(c)** and IFN-γ **(g)**, which have dual roles in inflammation, when compared to NT controls. (*n* = 7 animals per group, white bars – NT; gray bars – HT; error bars denote ±SD, * denotes *P* < 0.05).

In the brain, HT significantly decreased expression of pro-inflammatory cytokine IL-18, and cytokines with dual roles IL-6, and IFN-γ, 1 h after therapy was initiated (Figures 5c,f,g). While the differences did not reach statistical significance, HT also decreased expression of other pro-inflammatory cytokines IL-1α, MIP-1α, and TNF-α when compared to NT animals (Figures 5a,h,i). Interestingly, HT also decreased the expression of IL-10 and IL-13, which are considered anti-inflammatory mediators when compared to NT controls (Figures 5d,e). There were no significant differences in the expression of cytokines in the plasma of NT vs. HT treated animals, though there were trends toward decreased expression of IFN-γ, IL-1α, and IL-6 in HT treated animals (data not shown).

### HT Treatment Is Associated With Decreased Microglial Expression of C1q, C3, and C9

**FIGURE 6:**
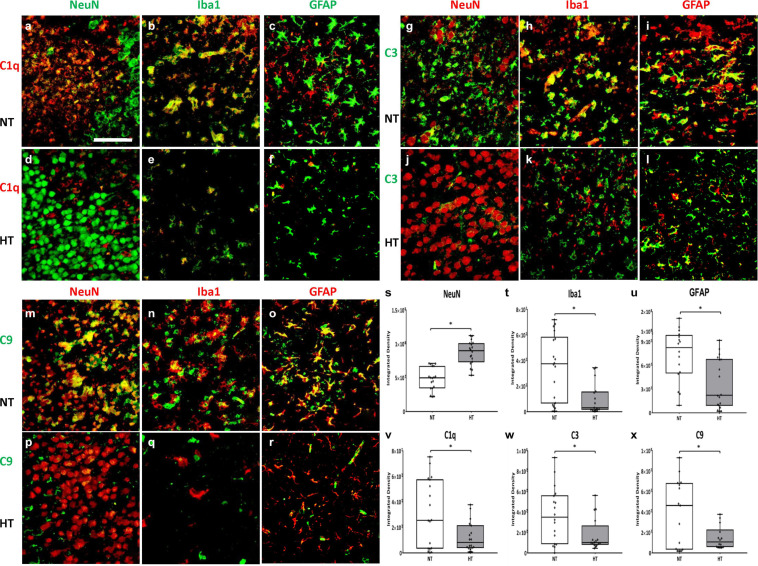
HT decreases microglial expression of C1q, C3 and C9. HT treated animals **(d,j,p)** demonstrated a higher neuronal density when compared to NT **(a,g,m)** animals **(s)**. HT animals showed decreased evidence of local inflammation as demonstrated by reduced microglial numbers and size **(e,k,q,t)** and reduced astrogliosis **(f,l,r,u)** when compared to NT animals **(b,c,h,i,n,o)**. C1q primarily co-localizes in the microglia **(b,e)**, with scant co-localization in neurons **(a,d)** and astrocytes **(c,f)**. C3 primarily co-localizes in the microglia **(h,k)** and astrocytes **(i,l)**, with scant co-localization in neurons **(g,j)**. C9 extensively co-localizes with neurons **(m,p)**, microglia **(n,q)** and astrocytes **(o,r)**. HT animals showed decreased expression of C1q **(v)**, C3 **(w)** and C9 **(x)** when compared to NT animals. [Ipsilateral hemispheres, cortex and hippocampus, 48 h after injury; *n* = 4 per group, 10 fields per brain, Magnification -40X; Green = NeuN **(a,d)**, C3 **(g–l)**, Iba1 **(b,e)**, GFAP **(c,f)**, C9 **(m–r)** Red = C1q **(a–f)**, NeuN **(g,j,m,p)**, Iba1 **(h,k,n,q)**, GFAP **(i,l,o,r)**; White bar – HIE, Gray bar – HT. Scale bars: 50 μm; error bars denote ±SD, * denotes *P* < 0.05].

HT treated animals (Figures 6d,j,p) demonstrated a higher neuronal density when compared to NT (Figures 6a,g,m) animals in the ipsilateral cortex and hippocampus (Figure 6s; *P* < 0.05 NT vs. HT). HT animals showed decreased evidence of local inflammation as demonstrated by reduced microglial numbers and size (Figures 6e,k,q) and reduced astrogliosis (Figures 6f,l,r) when compared to NT animals (Figures 6b,c,h,i,n,o) in the ipsilateral cortex and hippocampus (Figures 6t,u; *P* < 0.05 NT vs. HT). Contralateral hemispheres of the brain of NT animals showed scant Iba1 and GFAP expression, and robust NeuN staining (Supplementary Figure 1).

C1q primarily co-localizes in the microglia (Figures 6b,e), with scant co-localization in neurons (Figures 6a,d) and astrocytes (Figures 6c,f). C3 primarily co-localizes in the microglia (Figures 6h,k) and astrocytes (Figures 6i,l), with scant co-localization in neurons (Figures 6g,j). C9 extensively co-localizes with neurons (Figures 6m,p), microglia (Figures 6n,q) and astrocytes (Figures 6o,r).

Quantification of C1q in the ipsilateral cortex and hippocampus 48 h after initial insult shows significant reduction in C1q expression in HT animals when compared to NT animals (Figure 6v; *P* < 0.05 NT vs. HT). Similarly, HT animals showed decreased expression in C3 and C9 in the ipsilateral cortex and hippocampus 48 h after initial insult when compared to NT animals (Figures 6w,x; *P* < 0.05 NT vs. HT). Thus, HT treatment is associated with decreased local and systemic expression of pro-inflammatory complement effectors up to 48 h after initial injury.

### HT Treatment May Be Associated With Decreased C1q Binding on Cells Undergoing Apoptosis

**FIGURE 7:**
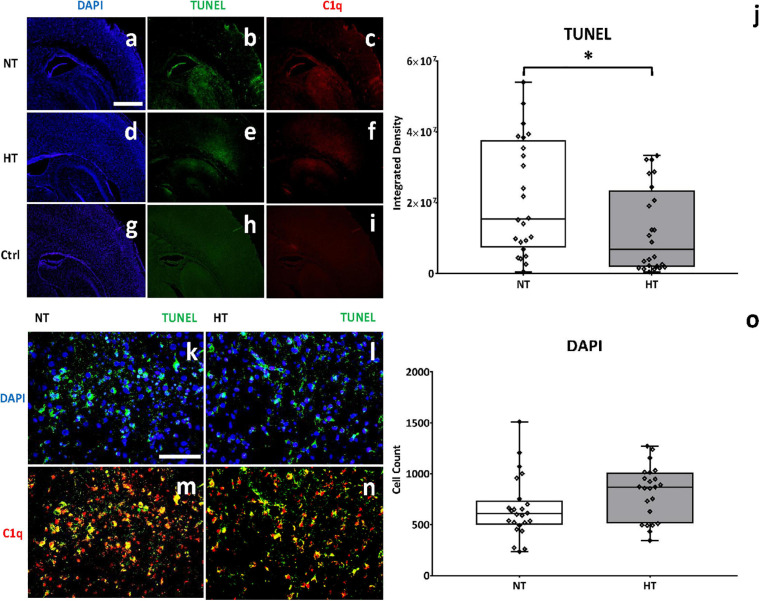
HT treatment may be associated with decreased C1q binding on cells undergoing apoptosis. NT animals **(a)** showed markedly reduced nuclear density in the cortex as demonstrated by DAPI staining when compared to HT **(d)** treated and Control/Sham **(g)** animals. Quantification of stained nuclei demonstrated improved cell counts in HT treated animals when compared to NT **(o)**. A marked increase in TUNEL positive cells **(b)** and c1q expression **(c)** was observed in NT animals, when compared to control animals **(h,i)**. HT treatment significantly reduced TUNEL positive cells **(e)** and C1q expression **(f)** when compared to NT animals. Higher magnification demonstrated decreased DNA fragmentation (green) in nuclei in HT treated animals **(l)** when compared to NT controls **(k)**. There was extensive co-localization of C1q in nuclei with DNA fragmentation in NT animals **(m)**, and a qualitative reduction in C1q/TUNEL co-localization in HT treated animals **(n)**. [Ipsilateral hemispheres, cortex and hippocampus, 48 h after injury; *n* = 4 per group, 10 fields per brain, Magnification 4X **(a–f)**, 40X **(g–j)**; 40X fields showing cortex; blue, DAPI; green, TUNEL; red, C1q; NT (white bar), HT (gray bar). Scale bars: 4X images 500 μm, 40X images 50 μm; error bars denote ±SD, * denotes *P* < 0.05].

To assess the effect of HT on cell death at 48 h after the initial injury, TUNEL staining was utilized to measure DNA strand breaks that are generated during apoptosis. Scant TUNEL staining was seen in brains of control/sham animals (Figure 7h). A marked increase in TUNEL positive cells was observed at 48 h in the cortex and hippocampus of the ipsilateral hemisphere of NT animals (Figure 7b). HT treatment reduced TUNEL positive cells in the ipsilateral hemisphere (Figure 7e). Quantification of DNA fragmentation using Image J (cortex and hippocampus) showed significantly fewer TUNEL positive cells in the HT group compared to NT (*P* = 0.04) treated animals (Figure 7j). NT animals showed markedly reduced nuclear density in the cortex as demonstrated by DAPI staining (Figure 7a) when compared to HT (Figure 7d) treated and Control/Sham (Figure 7g) animals. Quantification of stained nuclei also demonstrated improved cell counts in HT treated animals when compared to NT (Figure 7o). NT animals (Figure 7c) also had significantly more C1q expression in the cortex and hippocampus when compared to HT treated (Figure 7f) and control/sham (Figure 7i) animals. Higher magnification demonstrated widespread DNA fragmentation (green) in nuclei in the ipsilateral cortex of NT animals (Figure 7k). Treatment with HT (Figure 7l) reduced DNA fragmentation. There was extensive co-localization of C1q in nuclei with DNA fragmentation in NT animals (Figure 7m). There was a qualitative reduction in C1q/TUNEL co-localization in HT treated animals (Figure 7n). Thus, HT treatment was associated with significantly reduced apoptosis as measured by TUNEL staining. The decreased C1q/TUNEL co-localization in HT treated animals is consistent with the role of C1q in clearing apoptotic cells (Nauta et al., 2002).

## Discussion

This study demonstrates that treatment with HT is associated with decreased systemic and local expression of components of the classical complement pathway, HT treated animals have significantly decreased C1q, C3, and C9 expression in microglia and decreased neuronal apoptosis (Figure 1). HT also is also associated with decreased brain expression of several pro inflammatory cytokines, and systemic levels of the anaphylatoxin C5a, while the effect on other systemic cytokine expression is much more variable. However, HT treatment is also associated with decreased brain expression of anti-inflammatory and dual-role cytokines such as IL-6, IL-10, IL-13, and IFN-γ, and systemic C3a levels, a potentially neuroprotective complement effector. Lastly, HT treatment was associated with decreased neuronal apoptosis and decreased C1q binding in apoptotic cells. Our data demonstrate the extreme complexity of the immune response in neonatal HIE, and may help identify potential targets in the complement pathway for modulation that may enhance the neuroprotective effects of HT.

Hypoxic-ischemic encephalopathy is a characteristic ischemia-reperfusion injury, where tissue hypoxia and microvascular dysfunction are further complicated by subsequent reperfusion, activating innate and adaptive immune and inflammatory responses, which worsen tissue injury and destruction (Eltzschig and Eckle, 2011; Hassell et al., 2015). Complement activation in ischemia-reperfusion is primarily mediated by circulating natural antibodies (IgM) binding to neo-antigens expressed on the surface of hypoxia-stressed endothelial cells leading to classical complement pathway or lectin pathway activation (Figure 1). C1q is the initiator molecule of the classical complement cascade, and microglia are the predominant source of C1q in the brain (Fonseca et al., 2017). C1q was shown to be instrumental in microglial activation and neurodegeneration in a mouse model of retinal ischemia-reperfusion injury (Silverman et al., 2016). In our study, C1q was primarily expressed on microglia after HIE, and HT treated animals demonstrated reduced microglial population density as indicated by decreased Iba-1 immunoreactivity, and decreased C1q expression. While the decrease in C1q expression may be a direct suppressive effect of HT on the complement cascade, it is also possible that this decrease is a consequence of anti-apoptotic effects of HT (Luo and Pan, 2015). It is known that C1q is upregulated in response to ischemia as a protective mechanism, binding to neuronal blebs as well as to apoptotic neurons and significantly enhancing their phagocytosis by microglia. This facilitates rapid clearance of cell debris in a non-inflammatory fashion by microglial ingestion, and possibly preventing release of toxic intracellular contents such as glutamate from propagating injury to surrounding neurons. In our study, HT decreases neuronal apoptosis, and as a consequence the protective C1q response may be dampened. This is a recapitulation of a similar mechanism in the developing brain where C1q and microglia act in concert to tag supernumerary synapses for engulfment to facilitate synaptic organization and refinement (Perry and O’Connor, 2008). Furthermore, there is a transient increase in C1q expression in the neocortex postnatally, consistent with its possible role in eliminating excessive cortical connectivity (Coulthard et al., 2018).

We demonstrate that treatment with HT is associated with attenuation in the microglial expression of C1q, C3, and C9 in HIE. This may be one of the mechanisms by which HT mediates neuroprotection by quelling the opsonins and effectors of the complement cascade. The conventional understanding is that resident microglia are among the first cells to respond to ischemic brain injury, and exert neurotoxic functions by producing proinflammatory cytokines (IL-1β, IL-18, and TNF-α) and chemokines (MCP-1 and MIP-1α), which recruit leukocytes from the periphery (Lai and Todd, 2006; Weinstein et al., 2010). Recruited neutrophils, lymphocytes, dendritic cells and natural killer cells release free reactive oxygen species and proteases, increasing blood-brain barrier permeability and propagating injury to the ischemic tissue (Jin et al., 2010). Thus, the notion of microglial inhibition or depletion could be explored as a therapeutic approach in HIE. However, a cautious approach is warranted in the developing brain where microglia perform critical surveillance functions including synaptic pruning, modulation of neurotransmission, promotion of developmental apoptosis along with removal of apoptotic cells and secretion of growth factors for neuronal survival (Wakselman et al., 2008; Stephan et al., 2012; Nayak et al., 2014). During the first week of postnatal life, microglia produce IGF-1 to support survival and proliferation of layer V cortical neurons. This developmental function is seen again in aging or stroke, where production of IGF-1 by microglia promotes neurogenesis (Ransohoff, 2016). Microglial depletion in early postnatal life exacerbated neonatal neuronal damage after hypoxia-ischemia, possibly by reduced expression of IL-10 and TGF-β (Tsuji et al., 2020). The understanding of microglial diversity has evolved from the simplistic M1/M2 polarization paradigm to the discovery that microglia are a spatially and functionally heterogeneous community of cells where each subtype has unique properties not exclusively driven by their environment (Ransohoff, 2016; Stratoulias et al., 2019; Fisch et al., 2020). In a rat model of cortico-striatal hypoxia-ischemia, microglia in the sub-ventricular zone upregulate a heterogeneous pattern of genes related to pro- and anti-inflammatory as well as neurotrophic effects (Fisch et al., 2020).

Multiple studies have demonstrated an inconsistent effect of HT treatment on cytokine expression in HIE, likely due to the variability in disease expression. However, there do seem to associations between specific cytokines and long-term outcomes. Early systemic elevation of IL-1, IL-6, IL8, MCP-1, and VEGF were associated with abnormal neurological outcomes, and reduction in IL-6, IL-8, MIP-1α, and IL-10 over time was observed in infants with better outcomes (Jenkins et al., 2012; Chalak et al., 2014; Orrock et al., 2015; Perrone et al., 2018). In our study, while the decrease in brain expression of predominantly pro-inflammatory cytokines such as IL-18 likely accounts for the positive effects of HT, these effects may be tempered by the decrease in expression of possibly anti-inflammatory effectors such as IL-10, IL-13, C3a, and dual action effectors such as IL-6 and IFN-γ, which may have anti-inflammatory actions depending on the situation. Cytokine actions are pleiotropic and may have pro and anti-inflammatory effects in different contexts, and the deleterious consequences may be due to a protective response that spirals out of control (Rothaug et al., 2016). IL-18 levels were significantly increased in the NT group in a rat model of neonatal HIE (Hedtjärn et al., 2002). Treatment with HT was associated with attenuation in IL-18 levels and decreased microglial activation (Fukui et al., 2006). However, IL-18 expressed in the central nervous system also influences energy homeostasis and behavior (Fukui et al., 2006). Similarly dual roles of IL-6, IL-10, IFN-γ, and TNF- α in neuroinflammation depending on context have been described (McAdams and Juul, 2012). These inconsistencies in cytokine modulation by HT, and their inherent pleiotropy, may indicate that the utility of cytokines in HIE may be more as a biomarker than a therapeutic target. Collation of cytokine data from epidemiologic studies may assist in identifying correlations between cytokines expression and outcomes in HIE.

Inherent variability in disease manifestation makes development of therapeutic interventions for HIE a challenge. Current therapeutic strategies for management of neonatal HIE are restricted to therapeutic hypothermia (Tagin et al., 2012). Over the past several years, attempts to increase the effectiveness and broaden the scope of HT for HIE have not been successful (Shankaran et al., 2017). Moreover, HT may be ineffective or even harmful in the setting of hypoxia-ischemia sensitized by inflammatory conditions such as chorioamnionitis (Wintermark et al., 2010; Hassell et al., 2015). Most importantly, the incidences of death or major neurologic disability remain high at 40-55% despite treatment with HT (Papile et al., 2014). Adjunct therapies with non-specific mechanisms of action such as erythropoietin, melatonin, cannabinoids and autologous umbilical cord stem cells are in various phases of study, but as of yet, none have been approved for use in humans (Martinello et al., 2017). The ideal adjunct therapy would target inflammatory or reparative pathways that are sub optimally modulated by HT in a developmentally directed therapeutic window.

Complement inhibition as a therapeutic strategy in the growing brain is tricky since complement has multiple roles in brain development, neuroinflammation and healing. These developmental processes are precisely controlled by various regulators (Veerhuis et al., 2011). Complement has a multifaceted role in hypoxia-ischemia, likely owing to involvement both in pathological inflammation and in regeneration (Coulthard et al., 2018). During ischemia-reperfusion injuries such as HIE, the amplification of the complement cascade produces pro-inflammatory effector molecules such as C5a, altering the balance between activation and regulation, which can result in host tissue damage (Brennan et al., 2012). However, prolonged complement inhibition may interfere with its reparative role. In a mouse model of spinal cord injury, complement inhibition with a C5a receptor antagonist was protective in the acute phase, but deleterious in the post-acute phase of injury. This was due to the critical role that C5aR plays in creating a protective barrier through astrocyte proliferation (Brennan et al., 2015). In contrast, in acute situations like ischemia-reperfusion injuries, C3a prevents mobilization and accumulation of neutrophils, thus serving an anti-inflammatory function. However, in chronic disease models, C3a contributes to disease progression through its proinflammatory actions (Coulthard and Woodruff, 2015). Intranasal administration of C3a in an animal model of stroke improved functional outcomes, and ameliorated cognitive impairment in an animal model of neonatal HIE (Moran et al., 2017; Stokowska et al., 2017). While C1q deficiency was found to be neuroprotective in an animal model of neonatal HIE, C1q knockout mice display spontaneous absence seizures and reduced threshold for both spontaneous and evoked epileptiform activity (Chu et al., 2010).

The induction of C1q synthesis following injury is therefore consistent with a protective response facilitating clearance of apoptotic cells or cell debris, and promoting the resolution of inflammation before further damage ensues.

In our previous publication, we demonstrated that systemic C3a levels and brain C3aR expression were higher in the hypothermia group and hypothesized that neuroprotective effect of hypothermia may be mediated, in part, by increased systemic C3a (Shah et al., 2017). However, in the current study, we observed that that C3a levels are decreased after hypothermia treatment. To investigate further, we measured C3aR levels in the brain by western blot and immunofluorescence (Figure 5), and noted that the trends are consistent with the previous publication. C3a is generated at the convergence of all known complement activation pathways (the classical, alternate and lectin pathways). We would account for the discrepant C3a levels due to varying levels of generation of C3a via the alternative and lectin pathways. The role of C3a in HIE remains nebulous, and needs to be studied further. However, the consistently decreased levels of systemic C5a and C1q, and decreased C1q, C3 and C9 deposition in the brain in HT treated animals demonstrate that neuroprotection by these mechanisms is not dependent on C3a production. Thus, modulation on C3a and C3aR may be an avenue to bolster the neuroprotective effects of HT.

Our study has several limitations. The descriptive nature of the study precludes making definitive causal associations between HT treatment and decreased neuronal apoptosis secondary to attenuation of microglia expressed C1q. It is also possible that the decreased complement activation is a consequence of decreased neuronal damage in HT treated animals. However the link between hypoxia-ischemia and complement activation is well studied (Cowell et al., 2003; Ten et al., 2005; Rocha-Ferreira and Hristova, 2015; Shah et al., 2017), and the neuroprotective effects of hypothermia are well known (Shankaran et al., 2005, 2012). This descriptive study details the effects of HT treatment on complement expression, and contributes novel data providing a timeline for C1q, C3a, C5a expression, elucidating in detail the expression of complement components in microglia, neurons and astrocytes. It is reasonable to assume that one of the neuroprotective mechanisms of HT may be through complement modulation. Even though the study was powered adequately, larger sample numbers may have decreased variability in the cytokine data.

The need for a therapeutic adjunct to HT is urgent (Natarajan et al., 2018). While the net effect of therapeutic hypothermia is to quell the potentially harmful immune response in HIE, optimally targeting specific inflammatory pathways triggered by HIE may have a synergistic effect. Upstream inhibition of the classical complement pathway targeting C1q while intriguing, may be undesirable in the developing brain due to its multifaceted role in synaptic refinement, and increased risk of seizures, memory loss, and other cognitive deficits in C1q deficient mice (Chu et al., 2010). Instead, a more nuanced approach of C5aR antagonism in concert with C3aR agonist (intranasal C3a) in the acute phase of inflammation may be worthy of consideration as an adjunct to HT (Moran et al., 2017; Stokowska et al., 2017; Brandolini et al., 2019).

## Conclusion

In conclusion, in a clinically relevant model of neonatal HIE, our study reveals the myriad ways in which therapeutic hypothermia affect complement expression. While there is decreased systemic and microglial expression of C1q, decreased systemic C5a levels, and decreased microglial and neuronal deposition of C3 and C9, the observational nature of our study does not provide definitive mechanistic insights. However, to our knowledge, this is the first study to look at expression of complement components in neurons and glia in a model of neonatal HIE. HT is the standard of care treatment for neonatal HIE, and a carefully considered strategy of complement modulation may provide a robust additive effect to further improve neurodevelopmental outcomes in neonatal HIE.

## Data Availability Statement

The raw data supporting the conclusions of this article will be made available by the authors, without undue reservation.

## Ethics Statement

The animal study was reviewed and approved by the Eastern Virginia Medical School (EVMS) Institutional Animal Care and Use Committee.

## Author Contributions

TS, FL, WB, and HP designed the study and analyzed the data. TS, HP, CK, and FL performed the experiments. TS, WB, and FL wrote the manuscript. All authors read and approved the final manuscript.

## Conflict of Interest

The authors declare that the research was conducted in the absence of any commercial or financial relationships that could be construed as a potential conflict of interest.

## Supplementary Material

Supplementary Figure 1 Contralateral hemisphere in NT animals does not show evidence neuronal injury or gliosis. Contralateral cortex of NT brains stained for microglia **(A)**, astrocytes **(B)** and neurons **(C)**. There is scant staining for microglia and astrocytes, and robust staining for neurons in the contralateral hemisphere, confirming that there is no neuronal damage or gliosis. 20X magnification.

## Abbreviations

HIE: hypoxic-ischemic encephalopathy
HT: therapeutic hypothermia
NT: normothermia
i.p.: intraperitoneal
IRI: ischemia-reperfusion injury
RT: room temperature
TTC: 2,3,5-triphenyl-2H-tetrazolium chloride
TUNEL: terminal deoxynucleotidyl transferase dUTP nick end labeling
DAPI: 4’,6-diamidino-2-phenylindole
NBF: normal buffered formalin
CVF: Cobra-venom factor
PBST: phosphate-buffered saline with tween 20
NDS: normal donkey serum
SD: standard deviation
NGS: normal goat serum
C3aR: C3a receptor
C5aR: C5a receptor.
