# Supplementary figures and images for: Therapeutic Hypothermia Inhibits the Classical Complement Pathway in a Rat Model of Neonatal Hypoxic-Ischemic Encephalopathy
Source: Front Neurosci. 2021 Feb 12;15:616734. doi: 10.3389/fnins.2021.616734 (PMC7907466; doi:10.3389/fnins.2021.616734)

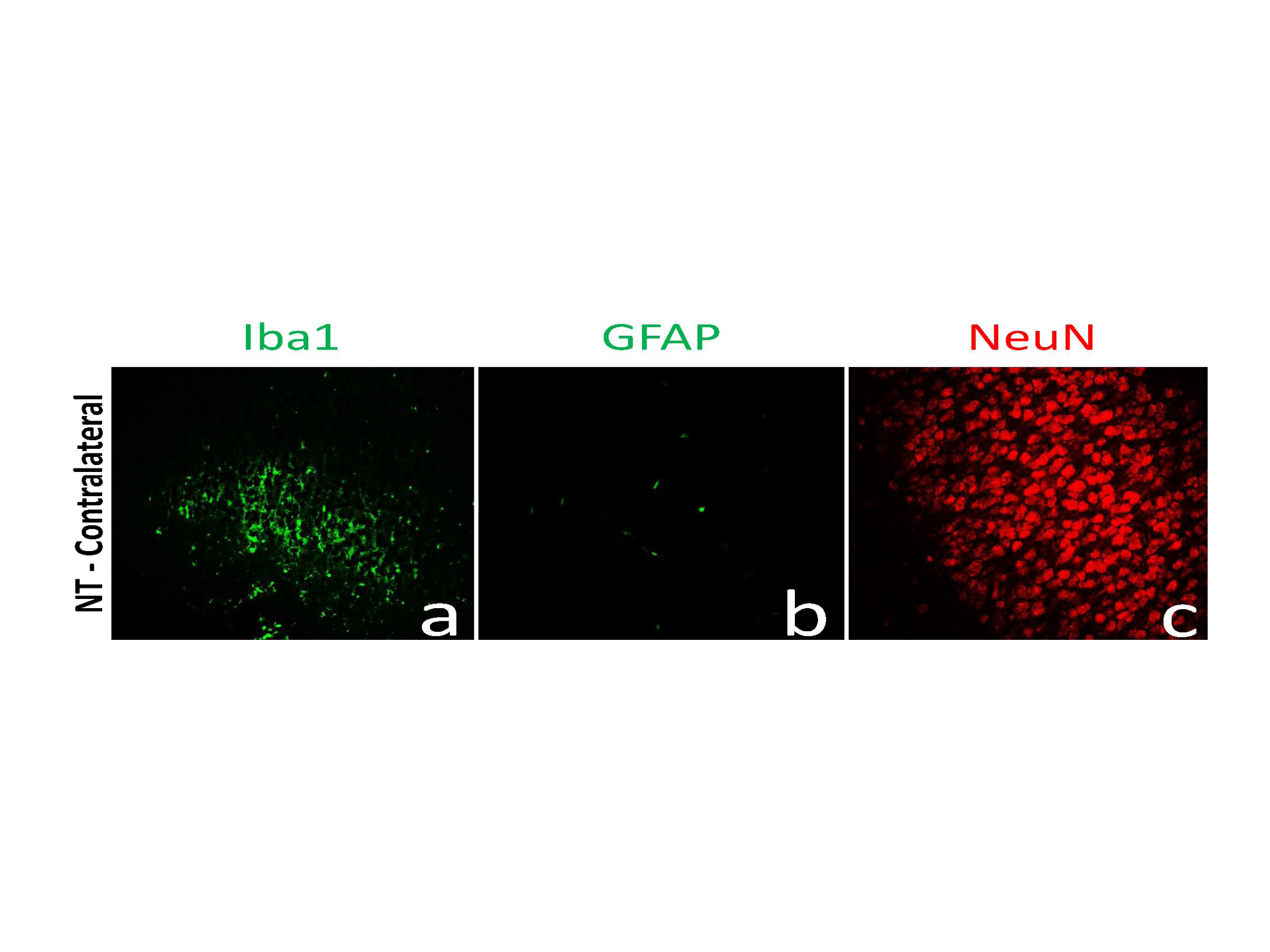

Supplement: Supplementary Figure 1 — Contralateral hemisphere in NT animals does not show evidence neuronal injury or gliosis. Contralateral cortex of NT brains stained for microglia (A), astrocytes (B) and neurons (C). There is scant staining for microglia and astrocytes, and robust staining for neurons in the contralateral hemisphere, confirming that there is no neuronal damage or gliosis. 20X magnification. [file Image_1.TIFF]
